# Thermal and Rheological Characterization of Aqueous Nanofluids Based on Reduced Graphene Oxide (rGO) with Manganese Dioxide Nanocomposites (MnO_2_)

**DOI:** 10.3390/nano12173042

**Published:** 2022-09-01

**Authors:** Felipe Lozano-Steinmetz, María Paz Ramírez-Navarro, Leonardo Vivas, Diego A. Vasco, Dinesh Pratap Singh, Carlos Zambra-Sazo

**Affiliations:** 1 Department of Mechanical Engineering, Faculty of Engineering, University of Santiago of Chile (USACH), Av. Lib. Bdo. O’Higgins 3363, Santiago 9170124, Chile; 2 Physics Department and Millennium Institute for Research in Optics (MIRO), Faculty of Science, University of Santiago of Chile (USACH), Av. Victor Jara 3493, Estación Central, Santiago 9170124, Chile; 3 Department of Mechanical Engineering, Faculty of Engineering, University of Talca, Curico 3460000, Chile

**Keywords:** reduced graphene oxide, nanofluids, thermal conductivity, viscosity, manganese dioxide

## Abstract

Nanofluids have become of interest in recent years thanks to their improved thermal properties, which make them especially interesting for microchannel heat sink applications. In this study, we prepared two aqueous nanofluids based on reduced graphene oxide (rGO) decorated with manganese dioxide (MnO${}_{2}$) at a concentration of 0.1 wt.%. The difference between the two nanofluids was in the preparation of the reduced graphene oxide decorated with MnO${}_{2}$. In the first case, the manganese salt was mixed with ascorbic acid before GO reduction with NaOH, and in the second case, the GO reduction with NaOH occurred under ascorbic acid. Ascorbic acid not only plays the role of a non-toxic and ecofriendly reducing agent but also acts as an important parameter to control the reaction kinetics. The structural, microstructural and spectral characterizations of the MnO${}_{2}$/rGO nanocomposite were conducted via X-ray diffractometry (XRD), Raman spectroscopy, FT-IR, TEM, SEM and EDS analyses. Moreover, the synthesized MnO${}_{2}$/rGO nanocomposites were utilized as nanofluids and their stability, thermal conductivity and rheological behaviors were studied. The thermal conductivity of the MnO${}_{2}$/rGO and MnO${}_{2}$AsA/rGO nanofluids was 17% and 14.8% higher than that of water for the average temperature range, respectively, but their viscosity remained statistically equal to that of water. Moreover, both nanofluids presented Newtonian behavior in the analyzed shear rate range. Therefore, both MnO${}_{2}$/rGO and MnO${}_{2}$AsA/rGO nanofluids are promising alternatives for use in applications with micro- and millichannel heat sinks.

## 1. Introduction

Graphene is a flat monolayer of carbon atoms compacted in a two-dimensional form (sp${}^{2}$ bonds), creating a honeycomb crystalline network, which fulfills a base function allowing the formation of other structures from graphitic materials [1]. Graphene’s crystalline network has interesting properties, such as high carrier mobility and long-range ballistic transport. The thermal conductivity of graphene near room temperature is in the range of 3000–5000 W/mK. Due to graphene’s excellent thermal conductivity, graphene-based materials have become a potential candidate for thermal management using nanofluids. Graphene-based nanomaterials are available in different nanostructures: multilayer graphene oxide (MLG), graphene quantum dots (GQDs), graphene nanoplatelets (GNP), graphene oxide (GO) and reduced graphene oxide (rGO) [2].

Graphene is a hydrophobic material and does not disperse in polar solvents. Consequently, graphene-based nanofluids’ stability is improved by adding surfactants. Following this strategy, Yu et al. [3] treated graphene nanosheets with sodium dodecylbenzenesulfonate (SDBS) and subsequently dispersed them in ethylene glycol (EG). Demirkir et al. [4] prepared nanofluids with graphene nano-flakes in DI water (0.1 wt.% to 2.0 wt.%), using polyvinyl pyrrolidone (PVP) as a surfactant. Other authors [5] synthesized magnetite Fe${}_{3}$O${}_{4}$-decorated GO nanosheets, dispersing them in water using sodium dodecylbenzene sulfonate (SDBS) as a surfactant. The maximum thermal conductivity enhancement observed was 11% for a nanofluid with 0.033 vol.%. Despite the remarkable stability improvements achieved by the use of surfactants in carbon-based nanomaterial nanofluids [6], at high temperatures, the surfactant may decompose, decreasing the performance of the nanofluids due to the formation of foam [7,8]. Foam generates thermal resistance, leading to a decrease in the thermal conductivity of the nanofluid [9,10]. Several chemical functionalization methods can provide long-term stability to nanofluids. Among them, researchers have used free-surfactant dispersion techniques, which generally involve treating the nanomaterial with an acidic or alkaline medium [11].

To obtain a hydrophilic structure of graphene, Ahmad Ghozatloo et al. [8] functionalized graphene nanosheets via an alkaline method (AFG). The nanofluid prepared by AFG dispersion in DI water exhibited enhanced thermal conductivity, reaching an increment of 17% with a concentration of 0.03 wt.%. According to the authors’ findings, the functional groups added to the graphene nanosheet (-COOK) improved the prepared nanofluids’ thermal conductivity compared to the effect of a pristine graphene nanosheet. In the same context, Lin et al. [12] treated a three-dimensional porous graphene-like (3D PG) material via an alkaline method. The functionalized 3D PG (f-3D PG) was characterized utilizing XPS spectroscopy, finding a trend for the formation of -COOH functional groups on its edge. The nanomaterial f-3D PG was dispersed in DI water, producing a suspension stable for more than one year and displaying a maximum enhancement in thermal conductivity of 97% at 60 ${}^{\circ }$C and a concentration of 0.07 wt.%. Graphene oxide is chemically modified graphene that is synthesized through exfoliation and oxidation, along with extensive oxidative modification of the basal plane [13]. GO consists of graphene sheets, which are synthesized directly from graphite powder using Hummers’ method [14]. The hydrophilic functional groups on the GO surface are conducive to good dispersion in polar solvents. GO is a monolayer material having high oxygen content with a variety of oxygenated functional groups, such as epoxide, hydroxyl, carboxyl and carbonyl groups [15]. The existence of these functional groups makes GO hydrophilic, and electrostatic stabilization explains the stability of GO in aqueous solutions. In graphene oxide, the number of carbon atoms bonded to oxygen atoms is greater than the number of intact sp${}^{2}$-hybridized carbon atoms, which modifies the properties of GO from pristine graphene. The existence of structural defects, poor stability, restacking and multilayer thickness can influence the features and surface area of GO. The insulating property of conventional GO also restricts its use in energy-related applications with nanofluids [16].

Shen et al. [17] reported that GO’s thermal conductivity is only 5% of that of pristine graphene. The thermal conductivity of GO nanofluids depends mainly on the particle size distribution and concentration of GO. Esfahani et al. [16] found that increasing the GO concentration from 0.01 wt.% to 0.1 wt.% resulted in 8.7% and 18.9% thermal conductivity enhancement at 25 ${}^{\circ }$C, respectively. Hajjar et al. [18] found that the thermal conductivity of water-based nanofluids with 0.05 wt.% and 0.25 wt.% of GO showed an increase of 14.75% and 47.57%, respectively. Xu et al. [19] studied the stability and the thermal conductivity of GO/water-based nanofluids in a concentration range of 0–1.5 wt.% and a temperature range of 20–60 ${}^{\circ }$C. The authors observed that the thermal conductivity of the nanofluid showed a maximum increase of 48.1%. Yu et al. [20] showed that GO enhances the thermal conductivity of distilled water by 30.2%, using a concentration of 5.0 vol.%. Akhavan-Zanjani et al. [21] measured the thermal conductivity of GO nanosheets/water-based nanofluids. The findings revealed a significant increment in the thermal conductivity (10.30%) with the addition of small amounts of GO nanosheets. Zhang et al. [22] fabricated GO by means of a modified Hummers’ and controlled reduced method (CRGO) and reported maximum thermal conductivity of 32.19% at 60 ${}^{\circ }$C for a concentration of 1.0 mg/mL. Noteworthy results were also obtained by Baby et al. [23], who showed that for graphene-based nanofluids (f-TEG), the augmentation in the thermal conductivity was 64%, with a volume fraction of 0.056% at 60 ${}^{\circ }$C concerning the pure water. Saeed Askari et al. [24] decorated GO with Fe${}_{3}$O${}_{4}$ and then suspended them in DI water. The authors indicated that the thermal conductivity reached a maximum improvement of 32% with 1.0 wt.% at 40 ${}^{\circ }$C and described nonlinear behavior for temperature and concentration. Selvaraj et al. [25] studied the behavior of nanofluids based on nanocomposites of graphene and Al${}_{2}$O${}_{3}$. The authors reported an increase in thermal conductivity, Nusselt number and heat transfer coefficient of 45%, 16% and 51.7%, respectively, with a concentration of 0.2 vol.%. Sarode et al. [26] prepared a nanofluid via the ultrasonic dispersion of GO/CuO nanocomposite in DI water. Functionalization considers the attachment of a CuO nanoparticles to O-H, C=O, C-O functional groups on the GO surface. The maximum thermal conductivity corresponded to a concentration of 0.03 vol.%, 12.47% and 26.78% greater than the nanofluids prepared with 0.02 vol.% and 0.01 vol.%, respectively.

Zhang et al. [22] proved, by using molecular dynamics simulation, that functionalized groups induce a reduction in the relative thermal conductivity of graphene due to the mass effect and structural deformation, which lead to graphene sheets losing their flat structure. Because these functional groups have the particularity of improving the hydrophilicity of the material and serve as active sites for chemical modification, it is possible to obtain a similar structure through the reduction of GO, rGO, which is also synthesized by Hummers’ method. From the reduction of GO, the stability of the nanofluids is critical to allow adequate heat transfer [27,28].

rGO is a two-dimensional, one-atomic, thin, layered material with a honeycomb structure, which is obtained by reducing the oxygen content of GO. Among the primary methods for reducing oxygen are thermal, chemical, microwave, photo-thermal, photo-chemical or microbial/bacterial methods [22]. The dispersion of rGO gives rise to more stable nanofluids. Zubir et al. [15] demonstrated that rGO nanofluids exhibit high robustness against thermally induced particle instability, using tannic acid as a reducer and stabilizer. Zhang et al. [22] studied controlled rGO nanofluids, which showed good dispersion stability with increased temperature and additive concentration. Said et al. [29] demonstrated that a hybrid mixture of functionalized carbon nanofibers/rGO nanofluid (0.04 vol.%) possessed outstanding stability by measuring the zeta potential.

The hybridization of rGO is a good alternative for the improvement of the thermal conductivity of nanofluids due to the effects of better stability. The addition of transition metal- based nanoparticles in reduced graphene (rGO) improves the stability of the rGO-based nanofluids since the oxidation of rGO is avoided [24,25]. Specifically, the addition of inorganic particles on rGO avoids the aggregation of graphene sheets and simultaneously improves the stability of the composite material, resulting from the MnO${}_{2}$/rGO vacancy interaction [30]. To follow an ecofriendly route for the synthesis of hybrid nanofluids, different methods for the functionalization of nanoparticles have been investigated based on the lipophilic modification procedure, which promises to obtain soluble and stable nanofluids in water [29]. Studies have shown that gallic acid is a natural compound rich in polyphenolic substances, which can be found in a variety of plants, such as ficus auriculate, green tea, grapes, palm dates and others, and they contribute to the stabilization and solubility of these nanoparticles [31,32]. Several authors have studied the behavior of these eco-friendly organic nanofluids, replacing the base fluid with an ecofriendly one or using nanoparticles generated in environmentally friendly ways. Nabeel Rashin and Hemalatha [33] studied the viscosity and stability of ZnO nanofluids in coconut oil at different concentrations. Sarafraz et al. [34] synthesized a biologically ecofriendly nanofluid, obtained from tea leaf extracts and aqueous silver nitrate, and high-quality silver nanoparticles, being a cheap and environmentally friendly process to produce silica nanoparticles from rice plants, to later synthesize water silica nanofluids. They studied the stability and thermal conductivity in the range of 25 to 55 ${}^{\circ }$C, observing an increase with respect to the base fluid of 33% for a volumetric concentration of 3%, being an alternative working fluid for thermal systems.

Zhang et al. [22] obtained significant thermal conductivity enhancements in rGO/DI water nanofluids, reaching a maximum enhancement of 32.19% at 60 ${}^{\circ }$C for an rGO concentration of 1.0 mg/mL. Accordingly, Kamatchi et al. [35] reported an enhancement in thermal conductivity of 10% for an rGO/DI water nanofluid (0.3 mg/mL) at 75 ${}^{\circ }$C. The rheology of rGO/water-based nanofluids has shown similar behavior to those prepared with GO, with Newtonian behavior at high shear rates [12,36]. Mehrali et al. [37] studied rGO and Ag-decorated rGO nanofluids, obtaining a viscosity increment of 22% for an rGO nanofluid, which is lower for Ag-decorated rGO nanofluids in the temperature range of 298-333 K. According to the study of Melaibari et al., the shear rate affects GO-CuO/water–EG hybrid nanofluids at concentrations of 0.8 and 1.6 vol.%. Therefore, rather than the shear rate, the base fluid is essential to explain the rheology of the nanofluids [38].

Decoration of the nanostructure surface of carbon-based nanomaterials is another approach to reduce the agglomeration tendency, maintaining their capability to improve thermal conductivity [39,40]. The decoration of nanostructures is possible since graphene oxide sheets have functional groups attached to their surfaces, generating synergic effects in several applications [33,35,41]. On the other hand, studies have demonstrated the functionalization of nanoparticles with gallic acid, e.g., graphene nanoplatelet nanofluids based on DI water functionalized by gallic acid, obtaining great stability and an improvement in thermal conductivity with respect to the base fluid of 24.18% for a concentration of 0.1 wt%. Mehrali et al. [37], functionalized rGO using polyphenols extracted from red wine, determining its chemical stability, wettability, electrical conductivity, heat capacity and thermal conductivity, which had an improvement over the base fluid of 45.1% for a volumetric concentration of 4%. According to this aspect, the procedure of lipophilic modification allows the participation of polyphenols from different natural sources that contribute to this functionalization; therefore, ascorbic acid is a potential candidate, as it can be used in the green reduction of graphene oxide, eliminating chemical routes of a toxic nature that generate environmentally harmful compounds, such as hydrazine, sodium borohydrate, hydrochloric acid and others, as indicated in the reduction via Hummers’ method [42,43].

In this study, MnO${}_{2}$ is considered as an alternative for the decoration of rGO to prepare stable nanofluids suitable for heat transfer applications, using a concentration of 0.1 wt%, since other authors have determined that rGO in the aqueous base at 0.1 wt% presents a great increase in its thermal conductivity while maintaining good stability [44]. The addition of inorganic MnO${}_{2}$ particles on rGO avoids the aggregation of graphene sheets and simultaneously improves the stability of the obtained nanofluids. Moreover, the hybrid rGO/MnO${}_{2}$ nanocomposite is achieved in an ecofriendly manner by reducing oxygen containing GO with ascorbic acid, which is a major component in all citrus fruits and vegetables. This study focuses on the physical analysis of the hybrid rGO/MnO${}_{2}$ nanocomposite and the thermal characterization of the prepared nanofluids, considering the effect of the hybridization technique of the nanocomposite on the stability, thermal conductivity and rheological behavior of the nanofluids.

## 2. Materials and Methods

### 2.1. Synthesis of MnO${}_{2}$/rGO Nanocomposites

en lThis section describes the synthesis of the materials for the nanofluids’ preparation. It is worth mentioning that all the chemicals were reactive-grade, provided by Sigma-Aldrich (Darmstadt, Germany). The details of the reactants and chemicals utilized are as follows; Mn(NO${}_{3}$)${}_{2}$ (SIGMA code: 288640-25G), C${}_{8}$H${}_{8}$O${}_{6}$ (SIGMA code: A92902-500G), H${}_{2}$SO${}_{4}$ (Sigma code: 258105), NaOH (MERCK code:106498.1000), potassium permanganate (Chemix code: 169708), H${}_{2}$O${}_{2}$ (Bios Lab Chile AG-0185).

#### 2.1.1. Synthesis of Graphene Oxide (GO)

GO was synthesized via the well-known Hummers’ method, with some modifications. For the synthesis of GO, 1 g of expanded graphite was mixed into 100 mL of H${}_{2}$SO${}_{4}$ and then 5 g of KMnO${}_{4}$ was slowly added to the beaker, which was kept in an ice bath to avoid any large rise in temperature due to exothermic reaction. The mixture was left under stirring for 2 h to oxidize the graphite surfaces, and the mixture was ultrasonicated for 1 h. After ultrasonication, it was kept under stirring for 3 days. Then, 600 mL of distilled water was added to the mixture and left under stirring for 15 min. Again, the solution was ultrasonicated for 2 h (85%), and 200 mL of distilled water was added again and stirred for 1 h. Finally, 60 mL of H${}_{2}$O was added under constant stirring for 1 h and then kept at room temperature to complete the reaction.

#### 2.1.2. Synthesis of Hybrid Nanoparticles (MnO${}_{2}$/rGO)

First, 2.88 g of Mn(NO${}_{3}$)${}_{2}$ was dissolved in 100 mL of distilled water and stirred for 10 min to create a homogenous solution. Then, 0.80 g of NaOH was mixed into the solution under constant magnetic stirring for 1 h. Furthermore, 100 mL of GO was added and the mixture stirred for an hour. The mixture was then placed on a hot plate heated up to 150 ${}^{\circ }$C, and the mixture was stirred for a further 30 min. Finally, 20 g of ascorbic acid C${}_{6}$H${}_{8}$O${}_{6}$ and 500 mL of distilled water were added to the heated mixture and left under constant temperature and stirring for 1 h.

#### 2.1.3. Nanocomposite Functionalization (MnO${}_{2}$AsA/rGO)

**Figure 1:**
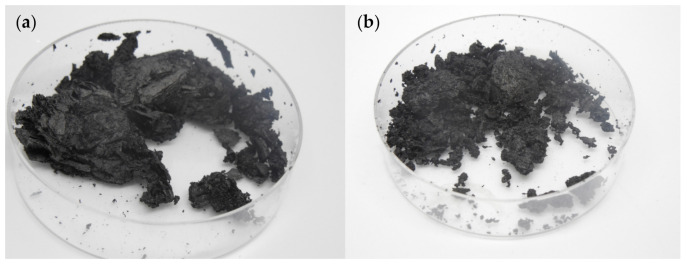
(**a**) Reduced graphene (rGO) oxide with manganese dioxide (MnO${}_{2}$) nanocomposite and (**b**) after functionalization with ascorbic acid.

First, 2.88 g of Mn(NO${}_{3}$)${}_{2}$ was dissolved in 100 mL of distilled water and stirred for 10 min to create a homogenous solution. Then, 0.5 g of ascorbic acid C${}_{6}$H${}_{8}$O${}_{6}$ was added and stirred for half an hour. Furthermore, 0.80 g of NaOH was added to the solution under constant magnetic stirring for 1 h. After this, 100 mL of the previously synthesized GO was mixed in and stirred for 1 h. The hot plate was heated up to 150 ${}^{\circ }$C and the mixture was stirred for a further 30 min. Furthermore, 20 g of ascorbic acid C${}_{6}$H${}_{8}$O${}_{6}$ was added to the heated mixture and left for an hour at the same temperature. Finally, 500 mL of distilled water was added and left under constant temperature and stirring for one hour. The obtained nanocomposite is shown in Figure 1.

The final solutions obtained from both functionalization methods contained 0.096 g of GO, and 2.88 g of Mn(NO${}_{3}$)${}_{2}$, equivalent to 0.844 g of Mn, i.e., a mass ratio of 0.11.

### 2.2. Characterization of the Nanocomposite

XRD analysis (XRD-Shimadzu 6000 powder diffractometer, Cu-K${}_{alpha}$ radiation, 40 kV and 30 mA) was performed for the nanocomposite characterization of rGO decorated with manganese dioxide. The morphology of the nanocomposites was analyzed by scanning electron microscopy (SEM Zeiss EVO MA|10) at 20 kV and a working distance of 8 mm. The images were captured with a secondary electron detector. The dispersed nanocomposite was characterized by transmission electron microscopy (TEM Hitachi, model HT7700 at 120 kV). For qualitative composition analysis, the energy-dispersive spectroscopy (EDS) method (Penta Precision, Oxford Instrument X-act) was used. Moreover, the structural analysis was performed by RAMAN spectroscopy, provided by the WITEC alpha 300 RA, equipped with 532 nm and 785 nm lasers, and Fourier transform analysis, using the IRTracer 100 spectrophotometer (Shimadzu).

### 2.3. Preparation of Nanofluids

**Figure 2:**
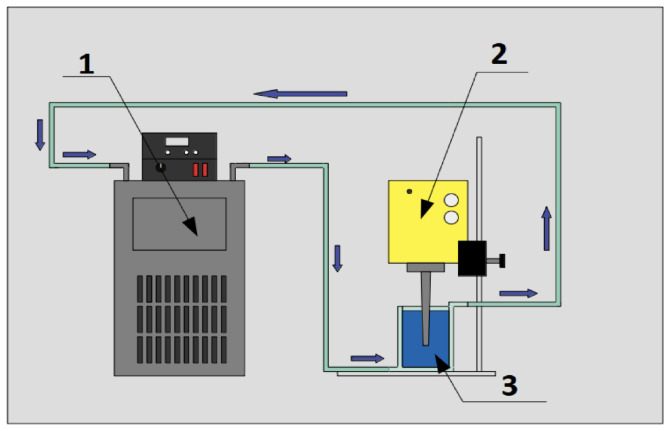
Experimental setup of the sonication process: (1) Refrigerated circulating bath, (2) ultrasonic probe Dr. Hielscher GmbH model UP50H and (3) sample in double jacketed vessel.

The nanofluids were synthesized using double-distilled water as a base fluid and the reduced graphene oxide decorated with MnO${}_{2}$ nanoparticles. The synthesis was carried out using the two-step method—specifically, the ultrasonic probe method—since the nanocomposite was in the form of a dry powder. For the synthesis of both nanofluids, a volume of 50 mL of base fluid was poured into a graduated cylinder, which was massed before being filled. Then, using an analytical balance (Radwag, model AS 82/220.R2), the amount of double-distilled water was massed. Then, the mixture was poured into a double-jacketed beaker that was connected to a refrigerated circulation bath (JSR, model 240 JSRC/13C) that maintained the water temperature at a constant level, always higher than 278.15 K, so that no condensate was generated, since this could affect the concentration. Equation (1) was used to determine the mass of the nanoparticles, using the mass of the base fluid and the required concentration:

(1) $$\;m_{np}=\frac{\varphi \cdot m_{bf}}{1-\varphi }$$

where $m_{np}$ corresponds to the mass of the nanomaterial, $m_{bf}$ to the mass of the base fluid and φ to the mass concentration. After determining the mass of the nanomaterial, the nanoparticle was massed using an analytical balance (Radwag, model AS 82/220.R2). Once the required mass of nanomaterial was obtained, they were incorporated into the double-jacketed glass and mechanically mixed with the help of a steel spatula, and then the ultrasonic cavitation process was started by means of an ultrasound probe. The probe used was a Dr. Hielscher GmbH ultrasound probe, model UP50H, which was used at its maximum amplitude of 180 μm, frequency of 30 kHz and power of 460 W/cm${}^{2}$ for 60 min, to obtain a homogeneous sample at 293.15 K. Figure 2 shows a diagram of the experimental setup used for the nanofluid synthesis process, by means of ultrasonic cavitation.

### 2.4. Thermal and Rheological Characterization of Nanofluids

In this section, we describe the thermal conductivity and viscosity measurement of both prepared nanofluids as a function of temperature. Thermal conductivity is essential for characterization since this property plays a fundamental role at the micro scale. Meanwhile, the rheology of the fluid is fundamental to understand the behavior of the nanofluid flow and the viscosity allows the calculation of the pumping power.

#### 2.4.1. Thermal Conductivity

**Figure 3:**
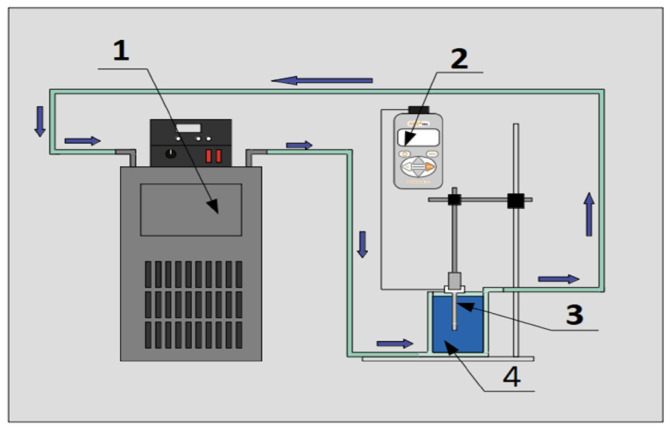
Experimental setup for thermal conductivity measurement. (1) Refrigerated circulating bath, (2) Decagon device property analyzer model KD2-Pro, (3) probe KS-1 and (4) sample in double jacketed beaker.

For the measurement of the thermal conductivity of the nanofluids and the base fluid, an experimental setup (Figure 3) with a thermal properties analyzer (KD2-Pro) and a KS-1 probe was used to carry out the transient linear heat source method (Equation (2)).

(2) $$\;\Delta T=\frac{q}{4\pi k}\left[-\gamma -ln\left(\beta \right)+\beta +\frac{\beta^{2}}{4}+...\right]$$

where $\beta =r^{2}/4\alpha t$, *q* is the heat dissipated per unit length of the wire, K is the thermal conductivity of the medium, γ is Euler’s constant (approximately 0.5572), α is the thermal diffusivity of the medium, *t* is the time of measurement, and *r* is the radius of the wire used.

**Figure 4:**
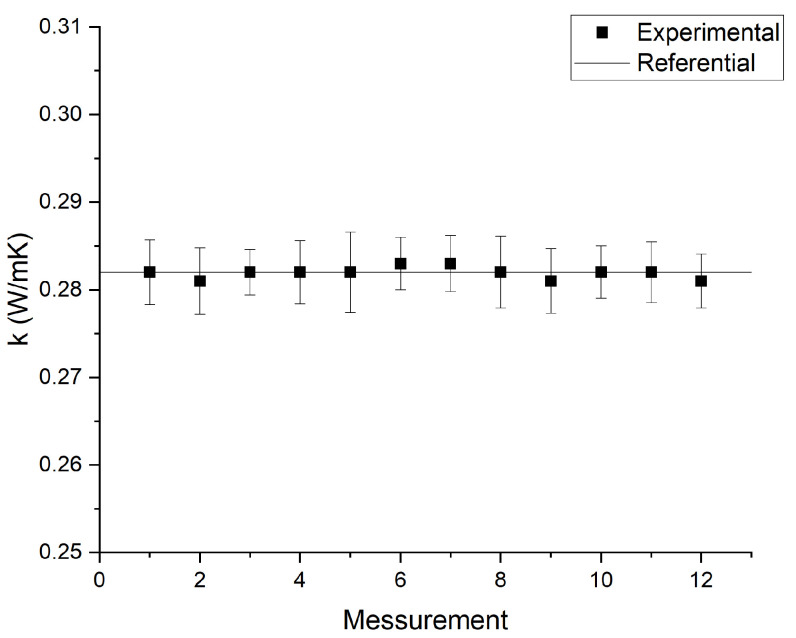
Traceability test results of KD2-Pro measurements. The error bars correspond to the KD2-Pro measurement error.

For calibration of the thermal conductivity measurement equipment, a traceability process was necessary. The traceability process of the thermal properties analyzer required 12 thermal conductivity measurements on glycerin (CAS 56-81-5), whose thermal conductivity is 0.282 W/mK at 293 K. The glycerin was supplied by the equipment manufacturer as standard material for the KS-1 probe. Figure 4 shows the obtained traceability results. A non-parametric Mann–Whitney test was performed to examine whether the experimental results obtained from the thermal properties analyzer differed from the reference.

The measurement of the thermal conductivity was carried out after sonication for 60 min, waiting for at least 5 min so that the sample reached a homogeneous temperature. Subsequently, the KS-1 probe was introduced into the sample in the center, to avoid natural convection during the measurement. Thermal conductivity measurements were performed for five temperatures, from 283.15 K to 303.15 K, with 5 K steps between each temperature, performing 6 measurements of the property per temperature level. Two thermal conductivity measurements were performed after the sonication was finished, with a waiting time of two minutes between them. Then, the sonication process was performed for 60 min again, to obtain 2 new measurements, a process that had to be repeated until 6 thermal conductivity measurements were reached for each temperature.

#### 2.4.2. Viscosity

**Figure 5:**
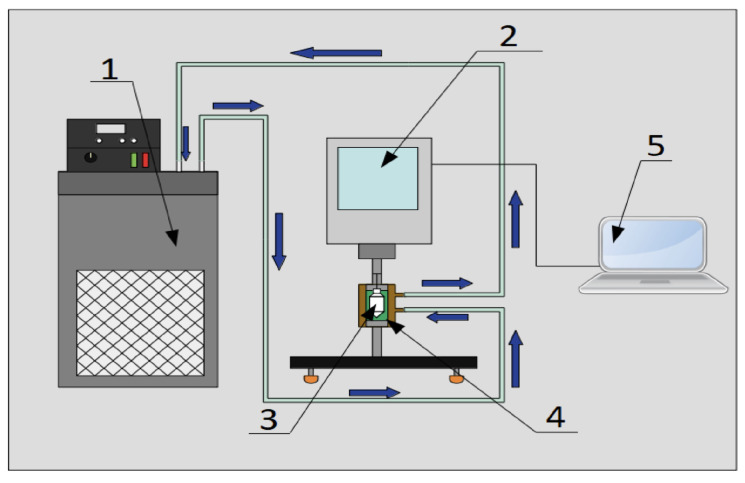
Experimental setup for viscosity conductivity measurement: (1) Refrigerated circulating bath, (2) Brookfield Viscometer model DV2T-LV, (3) spindle SC4-18, (4) sample and (5) computer with RheocalcT software.

To analyze the rheological behavior of the sample and the variation in the viscosity with respect to temperature, a Brookfield viscometer, model DV2T-LV, and spindle SC4-18 were used. The measurement process began with the assembly of the viscometer, which had to be leveled using the bubble level included in the equipment, to perform self-zeroing. Then, the instrument was connected to a computer with the “RheocalcT” software; then, the test parameters, such as RPM and measurement times, were entered into the software. Next, the spindle and the double-jacketed vessel, which was previously connected to a refrigerated circulation bath, were installed. Finally, the small sample adapter S4C-13R containing 7 mL of working fluid was connected to the double-jacketed vessel and the spindle was immersed in the sample so that the measurement could be performed Viscosity measurements were performed for eight temperatures, from 293.15 K to 328.15 K, with 5 K steps between temperatures, obtaining 5 measurements of the property per temperature level, with spindle rotation speeds up to 80 RPM and with steps of 10 RPM between levels. The setup can be seen in Figure 5.

In this study, the viscosity μ was obtained using Equation (3):

(3) $$\;\mu =\frac{\tau }{\dot{\gamma }}$$

where τ corresponds to the shear stress, and $\dot{\gamma }$ to the shear rate, which is calculated by the following equation:

(4) $$\;\dot{\gamma }=\frac{2R_{c}^{2}}{R_{c}^{2}-R_{b}^{2}}\omega$$

where *R*${}_{c}$ and *R*${}_{b}$ are the fluid container and spindle radius, respectively, and ω is the rotation speed in $s^{-1}$.

### 2.5. Stability Test

The stability of nanofluids was analyzed by UV–visible spectroscopy analysis with the Lambda 750 UV/Vis/NIR spectrophotometer from Perkin Elmer. Moreover, we assessed the nanofluids’ stability by visual inspection, collecting photographs during a period of 48 days. According to the Beer–Lambert law, there is a direct relationship between the absorbance *A* and the concentration of a sample (Equation (5)):

(5) $$\;A=\log_{10}\left(I_{0}/I\right)=\varepsilon cL$$

where $I_{0}$ is the intensity of the incident light at a given wavelength, *I* is the transmitted intensity, *L* the path length through the sample, *c* the concentration of the absorbing species and ε is the molar absorptivity.

## 3. Results and Analysis

The results of the structural, microstructural and spectral characterization of the synthesized rGO-MnO${}_{2}$ nanocomposites are analyzed first. Then, we present the results of the stability tests, and the thermal conductivity and rheology of the prepared nanofluids.

### 3.1. XRD

**Figure 6:**
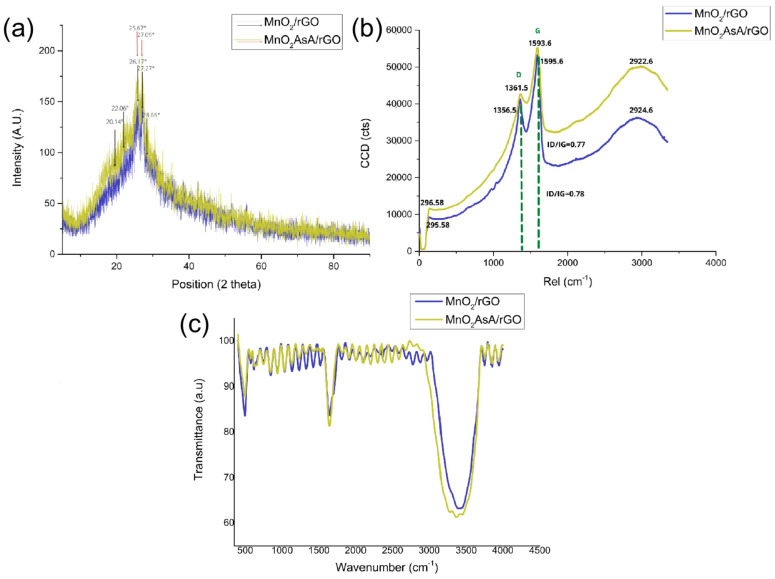
(**a**) XRD analysis of the nanocomposites MnO${}_{2}$/rGO and MnO${}_{2}$AsA/rGO, (**b**) Raman spectroscopy of MnO${}_{2}$/rGO and MnO${}_{2}$AsA/rGO nanocomposite particles and (**c**) FTIR spectra of MnO${}_{2}$/rGO and MnO${}_{2}$AsA/rGO nanofluids.

Figure 6a shows the X-ray diffraction pattern of the nanocomposite. The maximum peak of the rGO-MnO${}_{2}$ nanocomposite is observed at 2θ = 27.27${}^{\circ }$, which corresponds to rGO. It also appears for parameter (002) hkl in the form of the restoration of the graphitic structure in rGO after GO reduction with the removal of the oxygen residues between the graphitic layers. For MnO${}_{2}$, it is observed at angles of 2θ = 20.1${}^{\circ }$ and 28.6${}^{\circ }$, while Mn is observed at 2θ = 22.06${}^{\circ }$[45]. It can be observed that the MnO${}_{2}$/rGO has an overlapping of peaks at a reflection angle of 2θ = 26.1${}^{\circ }$, which corresponds to the (002) plane of rGO [46]. For the MnO${}_{2}$AsA/rGO nanocomposite, two maximum peaks are observed at 2θ = 25.67${}^{\circ }$ and 2θ = 27.09${}^{\circ }$, where the coupling of the MnO${}_{2}$/rGO signals occurs, in addition to the displacement of the position and intensity of these peaks, a product of the functionalization of the nanocomposite with ascorbic acid.

### 3.2. Raman Spectroscopy

Raman spectroscopy analysis was performed to obtain information on the molecular structure of the MnO${}_{2}$/rGO nanofluid. Figure 6b shows that there is a band of 1356.5 cm${}^{-1}$ and there is a high-intensity D-band present in the rGO and a lower-intensity G-band at 1595.6 cm${}^{-1}$. However, the ascorbic-acid-functionalized MnO${}_{2}$/rGO nanocomposite presents D- and G-bands at 1364.54 cm${}^{-1}$ and 1593.6 cm${}^{-1}$, respectively. Clearly, a decrease in band intensity is observed due to the reduction of oxygen functional groups. These changes in the width and intensity of the D- and G-bands indicate an increase in the structural disorder of the graphitic layers and arise from the creation of defects due to the reduction of oxygen-containing functional groups on the basal planes; in addition, the G-band is almost imperceptible due to the reduction of the oxygenated groups of graphene oxide [47,48,49].

The ratio of peak intensities D and G (ID/IG) indicates a ratio between amorphous and disordered carbon (sp${}^{3}$) with respect to graphitic carbon (sp${}^{2}$). These values are 0.78 (MnO${}_{2}$/rGO) and 0.77 (MnO${}_{2}$AsA/rGO), respectively. The slightly increased band intensity ratio in the MnO${}_{2}$/rGO nanofluid represents the reduction of oxygen functional groups in contrast to MnO${}_{2}$AsA/rGO [50]. It is likely that the premixing of ascorbic acid with the manganese salt slowed down the reaction kinetics and hence resulted in lower reduction of the GO composite. Finally, the bands at 295.58 and 296.58 cm${}^{-1}$correspond of the range of alpha MnO${}_{2}$ [51].

### 3.3. FT-IR

Figure 6c shows the functional groups’ interaction through FT-IR spectra of the MnO${}_{2}$/rGO and MnO${}_{2}$AsA/rGO nanofluids. The observed peaks at 3363.5 and 3394.5 cm${}^{-1}$, corresponding to O-H stretching vibrations, at 1647.2 cm${}^{-1}$ (C=O stretching vibrations), at 1600 cm${}^{-1}$ (skeletal vibrations from unoxidized graphitic domains), at 1200 cm${}^{-1}$ (C-O-C stretching vibrations) and at 1050 cm${}^{-1}$ (C-O stretching vibrations), are characteristic of rGO due to the remaining functional groups present on the graphene surface caused by incomplete reduction by the reducing agent. The removal of oxygen-containing groups during the reduction is confirmed from the decrease (almost disappearance) in the bands of C=O stretching, C-O-C stretching and C-O stretching. The relative decrease in the intensity of the O-H stretching band indicates that C-OH still exists, but in a lower proportion [52,53,54]. The sharp peaks at 497.63 and 493.68 cm${}^{-1}$ are attributed to the vibrations of the Mn–O bonds in MnOH [25,43].

### 3.4. SEM-TEM

**Figure 7:**
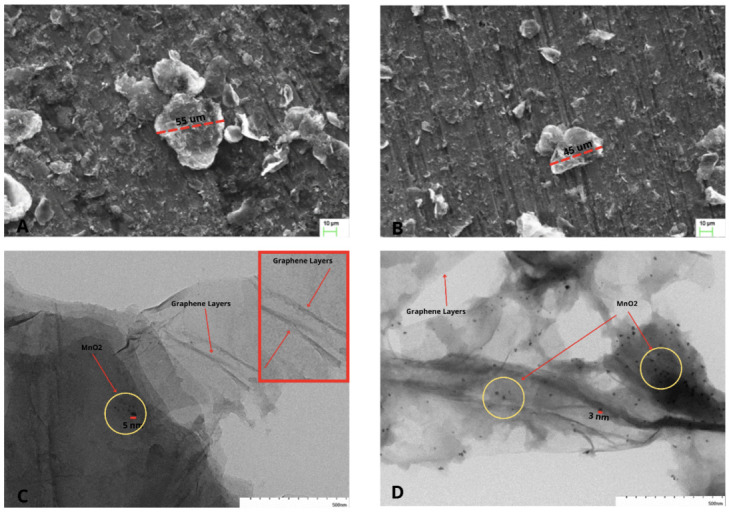
(**A**) SEM image of the MnO${}_{2}$/rGO nanocomposite; (**B**) SEM image of the MnO${}_{2}$AsA/rGO nanocomposite; (**C**) TEM image of the MnO${}_{2}$/rGO nanocomposite; (**D**) TEM image of the MnO${}_{2}$AsA/rGO nanocomposite.

The morphologies of the nanocomposites were analyzed by SEM. Figure 7A,B represent low-magnification SEM images of the MnO${}_{2}$/rGO and MnO${}_{2}$AsA/rGO nanocomposites, respectively, which show a large number of agglomerated flakes with a diameter of 10 μm and above. The magnified images of these flakes show thin graphene sheets and decorated MnO${}_{2}$ nanoparticles on the surface. The TEM micrographs in Figure 7C,D show very small-sized 5 nm MnO${}_{2}$ nanoparticles distributed over the surface of the reduced graphene oxide. Moreover, a higher number and better distribution of nanoparticles on the surface can be observed due to the role of ascorbic acid. On the other hand, the graphene sheets in Figure 7A,B have sizes in the order of micrometers for one of their dimensions, which makes rGO a 3D material, i.e., a material that exhibits nanocrystalline characteristics and nanostructure behavior, unlike MnO${}_{2}$, whose size is lower than 100 nm [55].

### 3.5. EDS (Energy-Dispersive Spectroscopy)

**Figure 8:**
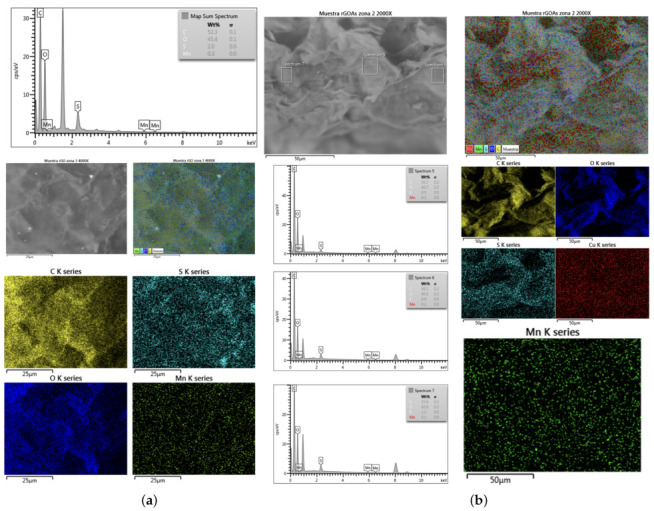
EDS analysis of the (**a**) MnO${}_{2}$/rGO (one zone) and (**b**) MnO${}_{2}$AsA/rGO (three zones) nanocomposites.

The EDS was performed (model SEM Zeiss EVO MA|10) to obtain a quantitative elemental analysis of the MnO${}_{2}$/rGO and MnO${}_{2}$AsA/rGO nanocomposites. In Figure 8a,b, the presence of the main elements of the nanocomposite, such as oxygen, carbon and manganese, can be observed. In addition, the presence of a small amount of sulfur in both samples may be due to the acid (H${}_{2}$SO${}_{4}$) used for the synthesis of GO by Hummers’ method. On the other hand, when comparing both figures, a decrease in the percentage of oxygen can be observed for the nanocomposite of MnO${}_{2}$AsA/rGO, due to the better reduction of the oxygen-containing functional groups due to presence of ascorbic acid before GO reduction. Figure 8a shows that the carbon and manganese content is 52.3 wt.% and 0.3 wt.% for the MnO${}_{2}$/rGO studied zone, similarly occurring in the three spectra of zone 2 of the MnO${}_{2}$AsA/rGO sample (Figure 8b), where the content of carbon and manganese amounts to 56 wt.% and 0.1 wt.%, respectively.

### 3.6. Stability of the Nanofluids

**Figure 9:**
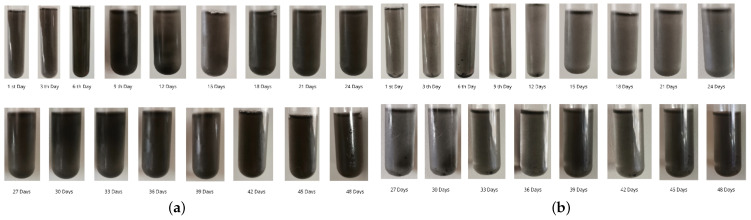
Stability of the nanofluids (**a**) MnO${}_{2}$AsA/rGO and (**b**) MnO${}_{2}$/rGO.

Initially, a qualitative study was carried out to obtain photographs of both nanofluids over a time period of 48 days. Figure 9 shows both nanofluids placed vertically, and it can be observed that both nanofluids are stable, presenting the minimum precipitation of nanoparticles in both cases. Nevertheless, we observed that the MnO${}_{2}$AsA/rGO nanofluid showed slightly higher stability compared to the MnO${}_{2}$/rGO nanofluid.

**Figure 10:**
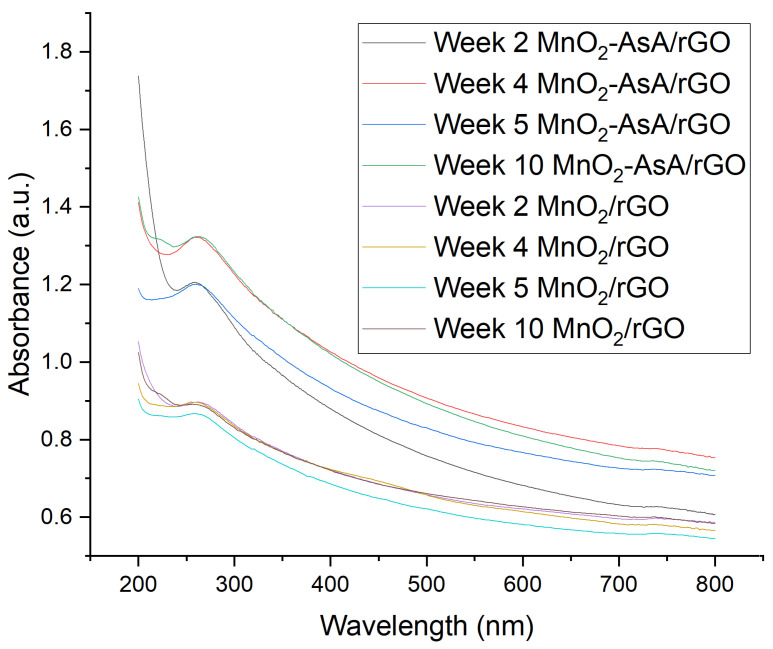
Absorbance versus wavelength of nanofluids during time.

An UV–Vis analysis complemented the qualitative study of the nanofluids’ stability. UV–Vis analysis confirmed the stability of both nanofluids over time since the absorbance presented minimal variation during the time period (Figure 10). The MnO${}_{2}$/rGO and MnO${}_{2}$-AsA/rGO nanofluids presented maximum absorbance variation of 0.030 and 0.122 UA, respectively.

The maximum absorbance values for the MnO${}_{2}$/rGO nanofluid could be found at the wavelength of 271 nm, while the average peak observed for the case of the MnO${}_{2}$AsA/rGO nanofluid throughout the four measurements carried out was found at the wavelength of 267 nm, presenting values similar to those presented by Bhanvase et al. [43] in their study (272 nm) and also that observed by Zhang et al. [56], showing a maximum absorbance at 264 nm. Likewise, the same behavior was observed in the absorbance peaks of rGO-SnO${}_{2}$ [57]. In comparison, graphene oxide nanofluids presented an absorbance peak at a lower wavelength of 236 nm [58] compared to those obtained in this study and in other studies with 404 decorated rGO; this difference is evidence of the successful interaction between rGO and the decorated nanoparticle.

### 3.7. Thermal Conductivity of the Nanofluids

**Figure 11:**
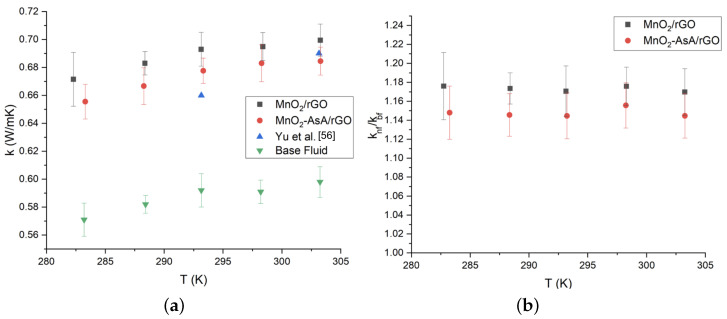
(**a**) Thermal conductivity of the nanofluids based on MnO${}_{2}$/rGO composites versus temperature and (**b**) relative thermal conductivity of nanofluids synthesized with of the nanofluids based on MnO${}_{2}$/rGO composites respect to the base fluid. The error bars (**a**) correspond to the measurements’ standard deviation and the propagation error (**b**) according to Equation (6).

In accordance with the measurements performed on the nanofluids synthesized with 0.1 wt.% of the nanocomposite MnO${}_{2}$/rGO, we observed an increase in the thermal conductivity of 17%. This improvement was reduced to 14.76% when MnO${}_{2}$AsA/rGO (0.1 wt.%) was used (Figure 11). Moreover, both nanofluids showed an increase in thermal conductivity with temperature, which can be seen in Figure 11. The obtained improvements in thermal conductivity are similar to those reported by Askari et al. [24], Vinodha et al. [59] and Yu et al. [57], which were 14%, 11% and 17%, respectively, for different decorated graphene-based nanofluids. Figure 11a also shows that our thermal conductivity results are quite similar to those reported by Yu et al. [57] for an aqueous nanofluid based on hydrogen-exfoliated graphene (HEG). Regarding the relative thermal conductivity, Figure 11b shows that both results exhibited the same behavior. A Shapiro–Wilk test with a 5% significance level was performed on the nanofluids to verify whether the use of ascorbic acid in the nanoparticle fabrication process influenced the thermal conductivity of the nanofluids. In this way, it was corroborated that for a concentration of 0.1 %wt, the mean recorded for the relative thermal conductivity of the nanofluid prepared with the nanoparticle without ascorbic acid did not differ from that recorded in those prepared using the nanoparticles with ascorbic acid, since the p-value was 0.6672, which allowed the null hypothesis of the test to be accepted. Therefore, the use of ascorbic acid in the manufacturing of nanoparticles does not generate differences in thermal conductivity.

As seen in Figure 11b, the error propagation equation was used to calculate the uncertainty of the relative thermal conductivity (Equation (6)):

(6) $$\frac{▵f}{\left|f\right|}=\sqrt{{\left(\frac{▵a}{a}\right)}^{2}+{\left(\frac{▵b}{b}\right)}^{2}}$$

Here, f=(a+▵a)/(b+▵b), *a*, *b* where arbitrary variables.

The MnO${}_{2}$/rGO nanofluids’ thermal conductivity is well described by a linear equation as a function of the temperature; the Equation (7) describe the model, and Table 1 presents the constants for both nanofluids [60].

(7) k=aT+b

The thermal conductivity of an aqueous nanofluid based on MnO${}_{2}$ with a concentration of 0.1 wt.% was predicted by using the model of Kumar et al. [61] (Equation (8)), which is especially valid for low concentrations considering the effects of particle size, particle volume fraction and temperature. This model allows the establishment of the relationship between the increase in thermal conductivity and the reduction in particle size, which is attributed to the high surface area to volume ratio of the nanoparticles, as well as to microconvection due to particle motion. Moreover, the increase in thermal conductivity with increasing temperature is explained by the intensification of the Brownian motion of the particles, which causes additional convective effects:

(8) $$\;\frac{k_{eff}}{k_{f}}=1+c\frac{2\cdot K_{B}T}{\pi \mu d_{p}^{2}}\frac{\varphi r_{f}}{k_{f}\left(1-\phi \right)}r_{p}$$

where $n_{p}$ is the diameter of the nanoparticle, $r_{p}$ the radius of the nanoparticle, $r_{f}$ the radius of the fluid particles, *c* is a constant (2.9 or 3) and K is the Boltzmann constant (1.318 × 10${}^{-23}$ J/K).

According to this model, the average thermal conductivity of the MnO${}_{2}$ nanofluid in the temperature range of 293–303 K is 0.59 W/mK, almost 15 % lower than the thermal conductivity of the rGO/MnO${}_{2}$ nanofluid in the same temperature range. Such a difference could be explained by the role of the high thermal conductivity of rGO in the heat conduction through its 2D structure. Table 2 shows the thermal conductivity of common nanoparticles, including metal oxides, graphene, GO and rGO. The thermal conductivity of rGO is even two orders of magnitude higher than that of metal oxides. Nevertheless, the improvements in the thermal conductivity of the rGO/MnO${}_{2}$ and MnO${}_{2}$ nanofluids are quite similar; therefore, microconvection and Brownian motion are the controlling mechanisms.

### 3.8. Rotational Rheology of the Nanofluids

**Figure 12:**
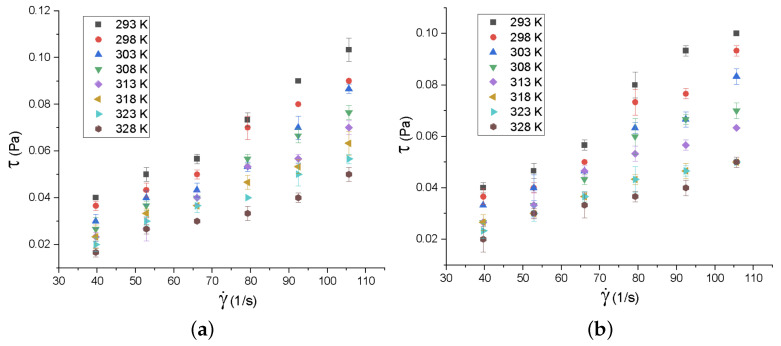
Rotational rheology of (**a**) MnO${}_{2}$AsA/rGO and (**b**) MnO${}_{2}$/rGO nanofluids. The error bars correspond to the measurements’ standard deviations.

The behavior of the shear stress with respect to the strain rate was also studied for both synthesized nanofluids, in the temperature range of 293 K to 328 K (Figure 12). To study the rheology of nanofluids, the Newtonian model was used, making a linear adjustment between the shear stress and the strain rate by means of the method of least squares. This model was used since, in the literature, graphene nanofluids (GO, graphene nanosheets, decorated GO, rGO and graphene nanoplatelets) exhibit Newtonian behavior [24,26,68,69,70,71].

A linear trend can be observed in Figure 12, which implies the Newtonian behavior of the samples. The R${}^{2}$ values are in the range of 0.9405 to 0.9882 for MnO${}_{2}$/rGO, while, for MnO${}_{2}$AsA/rGO, the R${}^{2}$ values range from 0.9098 to 0.9691. According to these results, the nanofluid based on MnO${}_{2}$AsA/rGO shows weaker Newtonian behavior, since the nanofluid based on MnO${}_{2}$/rGO presents an average R${}^{2}$ of 0.97 with a standard deviation of 0.0164, while the nanofluid based on rMnO${}_{2}$AsA/rGO presents an average R${}^{2}$ of 0.948 with a standard deviation of 0.0259. The average R${}^{2}$ of the MnO${}_{2}$/rGO nanofluid is 2.32% higher than that of the nanofluid based on MnO${}_{2}$AsA/rGO.

**Figure 13:**
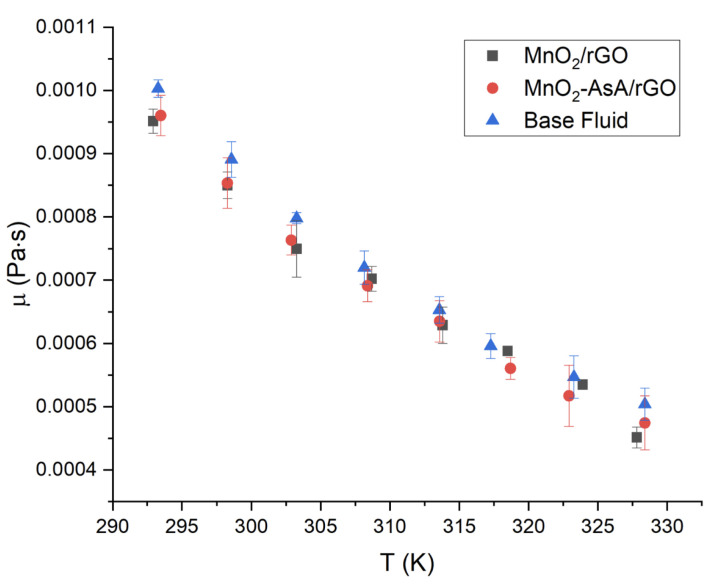
Dynamic viscosity versus temperature of MnO${}_{2}$/rGO nanofluids at a concentration of 0.1 wt.%. The error bars correspond to the measurements’ standard deviations.

The viscosity behavior with respect to temperature was studied in the range of 293–328 K, with 5 K steps between each levels. The viscosity values were obtained from the rheological study as the slope values from the linear fit, and the two nanofluids had similar 472 dynamic viscosity (Figure 13). The nanofluid based on MnO${}_{2}$/rGO presented a decrease in dynamic viscosity with respect to the base fluid of 4.62%, with a standard deviation of 3%. The viscosity of the nanofluid based on MnO${}_{2}$AsA/rGO was 4.60% lower than that of the base fluid, with a standard deviation of 1.09%. The reduction in both nanofluids’ viscosity regarding the base fluid can be attributed to the role of the movement of the nanoparticles or agglomerates in the transport of mechanical energy, which, as a product of Brownian motion, can have a rotational and a translational component, so that, when the space between the nanoparticles is larger than their dimensions (at low concentrations), the rotational and translational movements are not restricted by each other. Therefore, the shape of the nanoparticles influences the effective viscosity of the nanofluid [72].

A *t*-test with a 5% significance level showed that there were no significant differences in the viscosity of the three studied fluids: the MnO${}_{2}$/rGO-based nanofluid, the MnO${}_{2}$AsA/rGO-based nanofluid and water. Therefore, the viscosity of both MnO${}_{2}$/rGO and MnO${}_{2}$AsA/rGO nanofluids can be considered similar to that of water for thermal applications. Finally, the viscosity of both nanofluids as a function of temperature can be well described ($R^{2}=0.99$) by a quadratic model [73], presented in Equation (9). The corresponding parameters of the model are shown in Table 3.

(9) $$\;\mu =aT^{2}+bT+c$$

## 4. Conclusions

Stable nanofluids of MnO${}_{2}$/rGO with a concentration of 0.1wt% were synthesized, using bidistilled water as base fluid. We analyzed the effect of the addition of ascorbic acid during the functionalization process to form the nanocomposite MnO${}_{2}$AsA/rGO. The nanofluids are shelf-stable for more than one month.

The nanocomposite functionalized with ascorbic acid (MnO${}_{2}$AsA/rGO) showed a decrease in the oxygen-containing functional groups, mainly due to the action of ascorbic acid on the surface of the nanomaterial, which was also evident in the qualitative analysis in the higher homogeneity of the MnO${}_{2}$-decorated rGO in contrast to the nanocomposite MnO${}_{2}$/rGO.

The use of ascorbic acid for the synthesis and functionalization of MnO${}_{2}$/rGO nanocomposites is a sustainable and ecofriendly approach since it does not emit toxic components into the environment, unlike other synthesis routes. Moreover, the functionalization of MnO${}_{2}$/rGO with ascorbic acid gives rise to stable and promising nanofluids for applications with minichannel or microchannel heat sinks.

The nanofluids showed an average increase in their thermal conductivity with respect to the base fluid of 17% and 14.8% for MnO${}_{2}$/rGO and MnO${}_{2}$AsA/rGO, respectively. Both nanofluids exhibited Newtonian behavior in the studied strain rate range (40–106 s${}^{-1}$). Moreover, both nanofluids presented a viscosity difference of less than 0.5% and did not show significant differences in terms of water viscosity.

The studied nanofluids based on MnO${}_{2}$AsA/rGO have great potential to be implemented in heat removal systems, such as heat sinks of minichannels or microchannels, thanks to their high thermal conductivity, low viscosity and stability.

## Figures and Tables

**Table 1:** Fitted model parameters for the thermal conductivity of the nanofluids prepared with MnO${}_{2}$/rGO.

Nanofluid	a [W/mK${}^{2}$]	b [W/mK]
MnO${}_{2}$/rGO	$1.3156\times 10^{-3}$	$3.0278\times 10^{-1}$
MnO${}_{2}$AsA/rGO	$1.4876\times 10^{-3}$	$2.3716\times 10^{-1}$

**Table 2:** Thermal conductivity of the most commonly used nanoparticles to prepare nanofluids.

Nanomaterial	Thermal Conductivity [W/mK]
Graphene	5000 [62]
GO	0.5–18 [63]
rGO	1390–2275 [63]
MgO	8–29 [64]
CuO	18 [65]
TiO${}_{2}$	5.6 [66]
Al${}_{2}$O${}_{3}$	42.3 [65]
Fe${}_{2}$O${}_{3}$	80.4 [67]

**Table 3:** Fitted model parameters for the viscosity of the nanofluids prepared with MnO${}_{2}$/rGO.

Nanofluid	*a* [Pa· s/K${}^{2}$]	*b* [Pa· s/K]	*c* [Pa· s]
MnO${}_{2}$/rGO	$1.0327\times 10^{-7}$	$-7.7448\times 10^{-5}$	$1.4765\times 10^{-2}$
MnO${}_{2}$AsA/rGO	$1.9408\times 10^{-7}$	$-1.3431\times 10^{-4}$	$2.3653\times 10^{-2}$

## Data Availability

The data presented in this study are available on request from the corresponding author.
